# Chromosome-level genome assembly of *Utricularia aurea* Lour., a canivorious higher plant with minute genome

**DOI:** 10.1038/s41597-026-07285-1

**Published:** 2026-04-20

**Authors:** Jiaojun Yu, Shisheng Li, Hongjin Dong

**Affiliations:** Hubei Key Laboratory of Economic Forest Germplasm Improvement and Resources Comprehensive Utilization, Hubei Collaborative Innovation Center for the Characteristic Resources Exploitation of Dabie Mountains, Huanggang Normal University, Huangzhou, 438000 China

**Keywords:** Ecological genetics, Evolutionary genetics

## Abstract

*Utricularia aurea* Lour. is an aquatic herbaceous plant with yellow flowers and a unique insect-trapping mechanism, distributed in several regions of China and other Asian countries. In this study, we assembled a chromosome-scale genome of *U. aurea* with a size of approximately 180.31 Mb and a contig N50 length of 7.2 Mb. Using Oxford Nanopore long reads and Hi-C sequencing data, 99.99% of the assembled sequences were anchored onto 20 pseudo-chromosomes. We predicted a total of 33,365 protein-coding genes, of which 97.33% were functionally annotated using public databases including NR, GO, KOG, KEGG, and Swissprot. Additionally, we identified 117 rRNAs, 509 sRNAs and 382 tRNAs from the genome. The chromosome-scale *Utricularia aurea* genome will facilitate investigations into the genomic basis of its carnivorous adaptation, improve our understanding of the evolution history of carnivorous habits in *Utricularia*, and enrich research on the adaptive evolution of carnivorous plant genomes.

## Background & Summary

The genus *Utricularia*, within the Lentibulariaceae family, stands out as one of the most diverse groups of carnivorous plants, with over 200 species distributed globally across a wide range of habitats, from aquatic wetlands to terrestrial meadows and even epiphytic niches^1,2^. These plants have evolved highly specialized bladder-like traps, a characteristic feature enabling them to capture and digest small invertebrates efficiently, which compensates for nutrient deficiencies in their native environments^3,4^. Their genomes, despite often being relatively small, exhibit high gene density and remarkable plasticity, making *Utricularia* species prime models for studying the evolution of genome structure and function in response to ecological challenges^5,6^.

In recent years, genomic research on carnivorous plants, especially those in the *Utricularia* genus, has made significant strides. The sequencing of *U. gibba* revealed an 82 Mb genome with approximately 28,500 genes, characterized by a high proportion of coding DNA, minimal non-coding regions, and extensive gene loss and rearrangement events^5,7^. This compact genome structure was hypothesized to result from the selective pressure to reduce unnecessary genetic material in nutrient-poor habitats^6^. Conversely, *U. reniformis* has a much larger 304 Mb genome with 42,582 genes, where the expansion is mainly attributed to transposable element proliferation, particularly LTR-retrotransposons, and distinct post-whole-genome duplication (WGD) processes^8^. These studies have provided valuable insights into the genomic mechanisms underlying carnivory, such as the expansion of gene families related to prey capture, digestion, and nutrient uptake^7,8^.

Herein, we generated a chromosome-scale genome assembly of *U. aurea* using a combination of Oxford Nanopore long reads, Illumina short reads, and Hi-C sequencing data. Approximately 180.31 Mb of the genome was assembled, with a contig N50 length of 7.2 Mb. In total, 180.29 Mb (99.99%) of the assembled sequences were anchored onto 20 pseudo-chromosomes. This high-quality chromosome-scale genome of *U. aurea* will facilitate investigations into the genomic basis of its carnivorous adaptation, improve our understanding of the evolution history of carnivorous habits in *Utricularia* plants, and enrich research on the adaptive evolution of carnivorous plant genomes.

## Methods

### Plant materials and sequencing

**Fig. 1:**
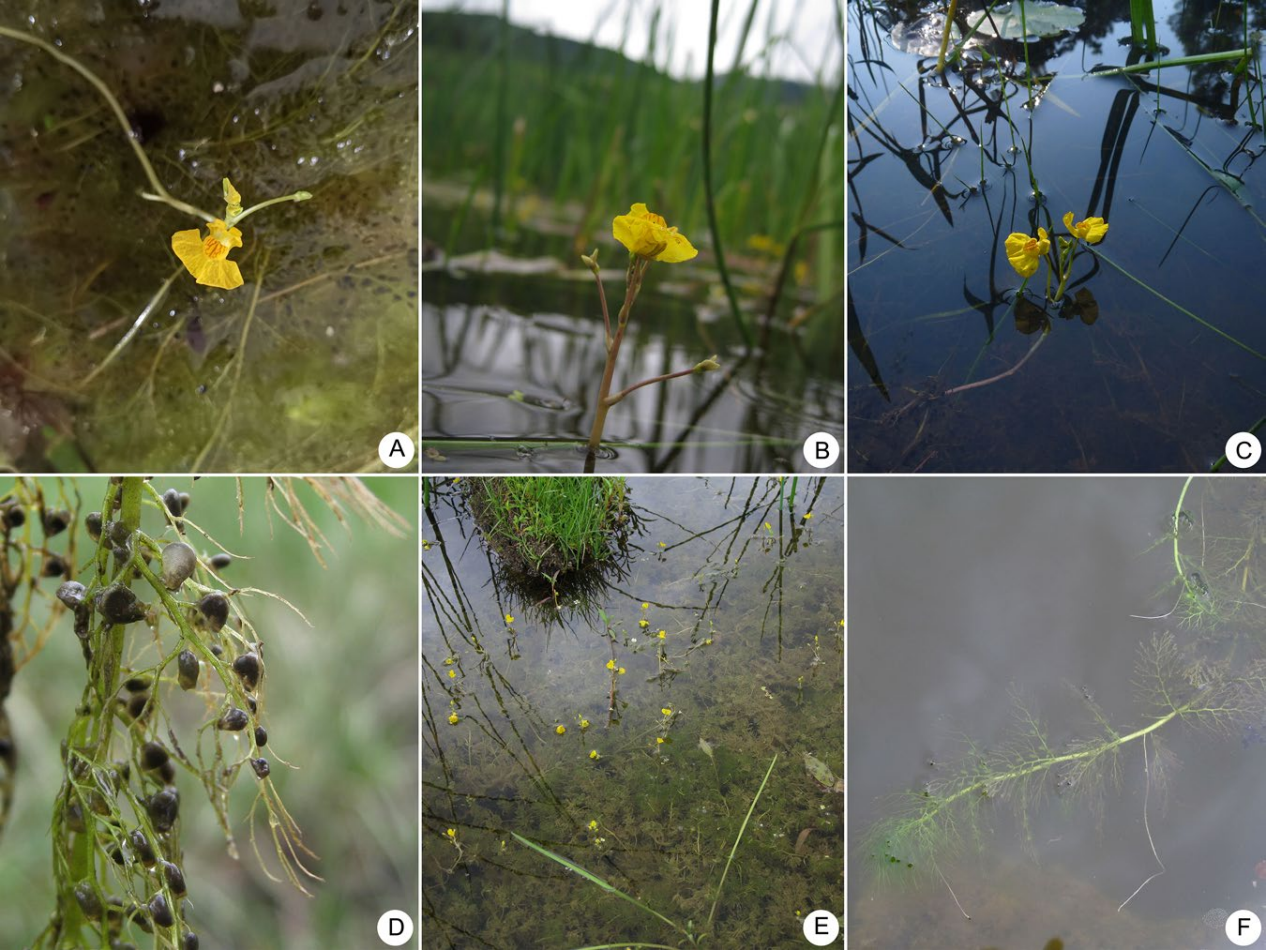
Morphological characteristics of *Utricularia aurea*. (**A**–**C**) Yellow flowers and living habitat; (**D**) Traps (black sac-like structures shown); (**E,F**) Wild habitat and individual plants.

The plant materials of *U. aurea* were collected from a wild pond in Xishui County, Huanggang City, Hubei Province (E 115.0892890°, N 30.4858364°, altitude 20 m) (Fig. 1). Continuous observations were conducted at this site for five years. All collected plant samples for sequencing were washed with ultrapure water, frozen in liquid nitrogen, and stored at −80 °C until further use. High molecular weight genomic DNA was prepared using the SDS method followed by purification with QIAGEN® Genomic kit (Cat#13343, QIAGEN) according to the standard operating procedure provided by the manufacturer. The DNA degradation and contamination of the extracted DNA was monitored on 1% agarose gels. DNA purity was then detected using a NanoDrop™ One UV-Vis spectrophotometer (Thermo Fisher Scientific, USA), of which OD260/280 ranging from 1.8 to 2.0 and OD260/230 was between 2.0–2.2. At last, DNA concentration was further measured by a Qubit® 3.0 Fluorometer (Invitrogen, USA). Total RNA was extracted from the collected fresh matiral for transcriptome sequencing. The qualified libraries were obtained after Poly-A RNAs enrichment, fragment, converted to cDNA, PCR-amplify, etc.

For Illumina short-read sequencing, DNA was randomly fragmented according to the manufacturer’s instructions. The qualified libraries were sequenced on the Illumina NovaSeq 6000 platform. In total, approximately 31.7 Gb of short-read sequencing data were generated, representing approximately 180.02 × genome coverage, which were subsequently utilized for genome survey analysis and sequence error correction during the genome assembly (Table 1). Oxford Nanopore sequencing was performed on a GridION X5 platform (Oxford Nanopore Technologies, ONT, UK) at Nextomics Biosciences Co., Ltd. (Wuhan, China)with the manufacturer’s instructions. A total of 51.5 Gb of pass reads (~292.47 × coverage) was generated (Table 1). For the Hi-C library construction, fresh tissue of *U. aurea* through a series of procedures from formaldehyde fixation to DNA purification, including nuclear isolation, enzymatic digestion, labeling, end repair and purification, etc. The final libraries were sequenced on the Illumina NovaSeq PE 150 bp platform, generating approximately 43.39 Gb of clean bases (~245.27× coverage) (Table 1).

**Table 1:** Genome assembly and assessment of *U. aurea* genome.

Assembly Item	Coverage Depth	Statistic/Values
Illumina sequencing (survey)	180.02 (X)	31.70 Gb
Nanopore sequencing	292.47 (X)	51.50 Gb
Illumina sequencing (Hi-C)	245.27 (X)	43.39 Gb
Estimated genome size (Mb) (Kmerfreq)		209.45
Estimated heterozygosity (%)(Kmerfreq)		0.7
Estimated genome size (Mb) (GenomeScope2)		200.54
Estimated heterozygosity (%)(GenomeScope2)		0.8
Scaffolds N50 (Mb)		7.14
Longest scaffold (Mb)		14.45
Number of scaffolds		56
Total length of scaffolds (Mb) (Nanopore assembled)		185.12
Contigs N50 (Mb)		7.2
Longest contig (Mb)		14.52
Number of contigs (Mb)		56
Total length of contigs (Mb) (H-ic assembled)		180.31
GC content (%)		39.49
Mapping with Illumina reads (%)		94.72
Accuracy of genome (%)		99.99
CEGMA assessment (%)		98.39
Completeness BUSCOs (%)		94.98
Complete single-copy BUSCOs (%)		53.72
Complete duplicated BUSCOs (%)		41.26

### Genome survey

**Fig. 2:**
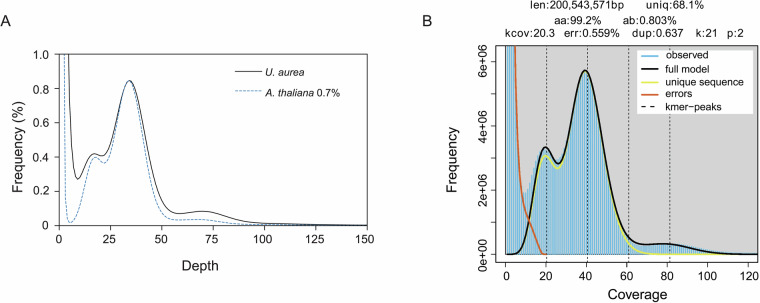
Genome survey of *U. aurea*. (**A**) K-mer frequency distribution curve (K = 17) using Kmerfreq. (**B**) K-mer depth distribution using GenomeScope2.

The raw short reads from Illumina were filtered using Fastp v.0.20.0 with default parameters to remove adapters, duplicates, and low-quality reads. Before genome assembly, the genome size and heterozygosity of *U. aurea* were estimated by k-mer analysis. Using Kmerfreq.^9^, genome size was evaluated based on the k-mer frequency distribution from clipping and statistical analysis (k-mer ≤ 19), and heterozygosity was inferred by integrating simulation data of *Arabidopsis thaliana* with varying heterozygosity levels and the frequency peak distribution of 17-mers. This analysis yielded an estimated genome size of *~*209.45 Mb and a heterozygosity rate of 0.70% (Fig. 2A and Table 1). In parallel, GenomeScope2^10^ estimated genome size as 200.54 Mb (assuming diploidy) with a heterozygosity rate of 0.8% (Fig. 2B and Table 1). Together, the results from the two programs indicate that the estimated genome size ranges from 200 to 210 Mb, with a heterozygosity rate of 0.7%–0.8%. These analyses confirm that the sequenced sample is free of contamination or essentially uncontaminated, and thus suitable for subsequent genome sequencing and assembly.

### *De novo* genome assembly of *U. aurea*

The Nanopore sequencers output FAST5 files containing signal data, and basecalling was first performed to convert the FAST5 files to FASTQ format with Guppy^11^. The raw reads of FASTQ format with a mean_qscore_template < 7 were then filtered resulting in pass reads. Subsequently, de novo genome assembly was performed using filtered ONT reads with NextDenovo v2.3.1^12^ under the parameters reads_cutoff:1k and seed_cutoff:35k. First, the NextCorrect module was used to correct the original long reads, yielding 3.8 Gb of corrected consensus sequences (CNS reads). Next, the NextGraph module (default parameters) was applied to assemble the corrected reads into a preliminary genome assembly of 184.35 Mb with a contig N50 of 7.14 Mb. To further improve the assembly accuracy, the contigs were polished using Nextpolish v1.3.0^13^ with three rounds of correction using ONT long reads and four rounds using Illumina short reads (both under default parameters), resulting in a final polished genome of 185.12 Mb with a contig N50 of 7.20 Mb (Table 1). To remove potentially redundant contigs and generate a final assembly, similarity searches were performed with the parameters “identity 0.8 –overlap 0.8”.

**Fig. 3:**
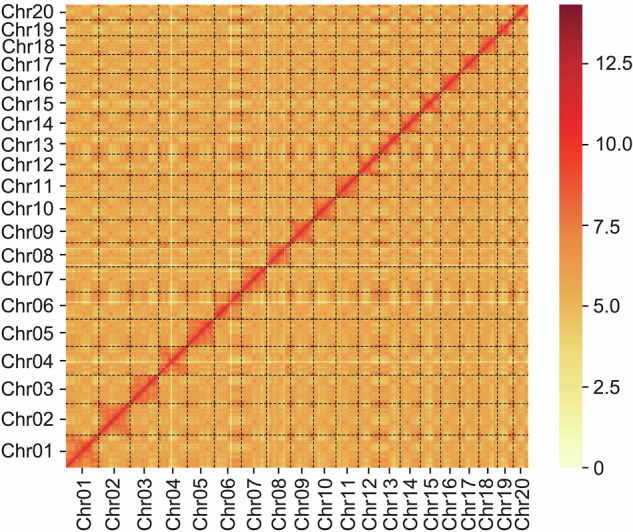
Hi-C contact map showing pairwise correlations among 20 pseudomolecules of *U. aurea*. Red intensity represents stronger Hi-C interactions.

For the Hi-C raw data, we used Fastp 0.12.6^14^ to filter and remove low-quality sequences (quality scores < 20), adaptor sequences and sequences shorter than 30 bp. The high-quality Hi-C reads were subjected to obtain scaffolds using bowtie2 (v2.3.2)^15^ and HiC-Pro (v2.8.1)^16^. Then scaffolds used to cluster, order and orient the contigs into pseudo-chromosomes using LACHESIS^17^, followed by manually adjusted based on chromosome interaction patterns. Finally, we assembled the final chromosome-scale genome of *U. aurea*, which is 180.31 Mb with a contig N50 of 7.2 Mb (Table 1). A total of 180.29 Mb (99.99%) of the sequences were successfully anchored to the 20 pseudo-chromosomes, and the chromosomes were numbered according to the order of chromosome length (Fig. 3, Supplementary Table S2).

### Repeat elements annotation

We first annotated the tandem repeats (TR) using software GMATA v2.2^18^ and TRF v4.07b^19^ with default parameters, where GMATA identifies the simple repeat sequences (SSRs) and TRF recognizes all tandem repeat elements in the whole genome. Transposable elements (TE) in the *U. aurea* genome were then identified using a combination of ab initio and homology-based methods. Briefly, an ab inito repeat library for *U. aurea* was first predicted using MITE-hunter^20^ and RepeatModeler v1.0.11^21^ with default parameters, in which LTR_FINDER, ltr_harverst and LTR_retriver were also be included for the genome. The obtained library was then aligned to TEclass Repbase (http://www.girinst.org/repbase) to classify the type of each repeat family. For further identification of the repeats throughout the genome, RepeatMasker 1.331^21^ was applied to search for known and novel TEs by mapping sequences against the de novo repeat library and Repbase TE library. Overlapping transposable elements belonging to the same repeat class were collated and combined. Totally, 46.23 Mb (25.64%) repetitive sequences were identified in the *U. aurea* genome. In the most abundant type of transposable elements (TEs, 23.08%), long terminal repeat (LTR) elements are the most predominant (13.59%), while LINEs (long interspersed nuclear elements) and SINEs (short interspersed nuclear elements) are relatively fewer (1.34% and 0.2%, respectively). A total of 11,452 simple sequence repeats (SSRs) were identified, accounting for 0.09% of the *U. aurea* genome (Table 2).

**Table 2:** Information of repeats elements of *U. aurea* genome.

Class	Type	Number of elements	Length of sequence (bp)	Percentage of sequence (%)
Class I	LINE	11,290	2,407,979	1.34
	LTR	65,718	25,157,194	13.95
	SINE	3,488	353,889	0.2
Class II	DNA	58,297	13,022,393	7.22
	MITE	632	61,030	0.03
	RC	1,867	617,005	0.34
Total TEs		141,292	41,619,490	23.08
Total TRs	Tandem repeat	20,482	1,097,909	0.61
	SSR	11,452	154,318	0.09
Other		221	46,164	0.03
Unknown		19,236	3,426,966	1.9
Simple repeats		322	42,670	0.02
Low complexity		4	981	0
Total Repeats		181,557	46,234,180	25.64

### Gene prediction and function annotation

Three independent approaches, including ab initio prediction, homology search, and reference guided-transcriptome assembly, were used for gene prediction. In detail, GeMoMa v1.6.1^22^ was used to align the homologous peptides from related species (*Capsicum annuum*, *Citrus sinensis*, *Daucus carota*, *Ipomoea triloba*, *Nymphaea colorata*, *Olea europaea* and *Sesamum indicum*) to the assembled *U. aurea* genome and then got the gene struture information, which was homolog prediction. For RNAseq-based gene prediction, filtered mRNA-seq reads (totally 34.4 Gb clean data from Illumina NovaSeq 6000) were aligned to the reference genome using STAR 2.7.3a^23^ (defaultment). The transcripts were then assembled using stringtie and open reading frames (ORFs) were predicted using PASA v2.3.3^24^. For de novo prediction, RNA-seq reads were de novo assembled using StringTie v1.3.4d^25^, and analyzed with PASA v2.3.3^24^ to produce a training set. Augustus v3.3.1^26^ with default parameters was then utilized for ab initio gene prediction with the training set. Finally, EVidenceModeler v1.1.1^26^ (EVM) was used to produce an integrated gene set of which genes with TEs were removed using TransposonPSI package (http://transposonpsi.sourceforge.net/) and the miscoded genes were further filtered. Untranslated regions (UTRs) and alternative splicing regions were determined using PASA v2.3.3^24^ based on RNA-seq assemblies. We retained the longest transcripts for each locus, and regions outside of the ORFs were designated UTRs. Finally, a gene set consisting of 33,365 genes was annotated in *U. aurea* genome. (Supplementary Table S3).

To obtain the ncRNA (non-coding RNA), two strategies were used: searching against database and prediction with model. Transfer RNAs (tRNAs) were predicted using tRNAscan-SE v2.0^27^ with eukaryote parameters. Infernal cmscan v1.1.2^28^ was used to search the Rfam database^29^ to identify ribosomal RNA (rRNA), small nuclear RNA (snRNA), microRNA (miRNA), small nucleolar RNA (snoRNA, eg. spliceosomal) and others. The rRNAs and their subunits were predicted using RNAmmer v1.2^30^. In sum, a total of 117 rRNAs, 509 sRNAs (small RNAs, including 304 snRNAs, 114 miRNAs, and 76 spliceosomal), and 382 tRNAs were annotated (Table 3). Gene function information, motifs and domains of their proteins were assigned by comparing with public databases including SwissProt, NR, KEGG, KOG, and Gene Ontology. The putative domains and GO terms of genes were identified using the InterProScan program v5.32-71.0^31^ with default parameters. For the other four databases, BLASTp v2.7.1^32^ was used to compare the EvidenceModeler-integrated protein sequences against the four well-known public protein database with an E-value cutoff of 1e−05 and the results with the hit with lowest E value was retained. As a concatenated of the five database searches results, 32,475 (97.33%) genes of the *U. aurea* genome were successfully annotated (Table 4).

**Table 3:** Non-coding RNAs annotation of *U. aurea* genome.

Type Category	Type	Copy Number	Average Length(bp)	Total Length(bp)	Percentage of sequence(%)
rRNA (117)	18S	6	1,846.50	11,079	0.0061
	28S	8	4,443.50	35,548	0.0197
	5.8S	1	156	156	0.0001
	5S	102	112.15	11,439	0.0063
sRNA (509)	snRNA	304	99.53	30,258	0.0168
	miRNA	114	118.21	13,476	0.0075
	spliceosomal	76	145.89	11,088	0.0061
	other	15	249.07	3,736	0.0021
tRNA	tRNA	382	74.68	28,529	0.0158

Note: rRNA (ribosomal RNA), sRNA (small RNA), tRNA (transfer RNA).

**Table 4:** Gene function annotation of *U. aurea* genome.

Database	Gene Number	Annotation Ratio (%)
KOG	18,880	56.59%
KEGG	14,021	42.02%
NR	32,386	97.07%
SwissProt	27,995	83.91%
GO	20,555	61.61%
Overall annotated gene	32,475	97.33%
Total gene	33365	—

## Data Records

The raw data of Nanopore, Illumina and Hi-C sequencing (Illumina data) were submitted to the National Center for Biotechnology Information (NCBI) SRA database with accession number SRR34843000^33^, SRR34844152^34^, SRR34844153^35^, SRR34844667- SRR34844670^36–39^ under BioProject accession number PRJNA1289348. The assembled genome and anotated information had been deposited at GenBank with accession number JBPYBC000000000^40^. The assembled genome and annotation results have been deposited in the figshare database^41^.

## Technical Validation

### Evaluation of the assembled genome

To assess the quality of the final genome assembly, Illumina short reads were aligned to the draft genome using BWA 0.7.12-r1039^42^ with default parameters. We used samtools v1.4^43^ to analyze the alignment results, which showed an alignment rate of 94.72% (Table 1). Subsequently, we used bcftools v1.8.0^44^ for SNP and Indel detection to count heterozygous and homozygous sites (homozygous sites were considered genomic errors), and the results indicated a single-base accuracy of 99.99% (Table 1). We further evaluated the completeness of the final genome assembly using CEGMA v2^45^ and BUSCO v4.0.5^46^. The CEGMA database contains 248 conserved core eukaryotic genes, and our assembled genome included 244 of these genes, representing 98.39% completeness (Table 1). Using the Embryophyte database of embryophyta_odb10, BUSCO analysis identified 1,533 out of 1,614 expected embryophyte genes (94.98%) (Table 1). Furthermore, the Hi-C assembly achieved high quality with a QV score of 33.93 estimated by Merqury v1.3^47^, exceeding the widely accepted threshold of 30 for high-quality genomes (Supplementary Table S2). Notably, QV scores within each pseudo-chromosomewere all above 31.9, further supporting the high consensus accuracy of the assembly (Supplementary Table S2).

### Evaluation of the gene annotation

BUSCO v4.0.5^46^ was used to evaluate the gene set obtained from the prediction. The results indicated that approximately 93.31% of the complete gene elements were identified within the annotated gene set (Table S4). Additionally, we compared the genetic information of *U. aurea* with its closely related taxa (*Capsicum annuum*, *Citrus sinensis*, *Daucus carota*, *Ipomoea triloba*, *Nymphaea colorata*, *Olea europaea*, and *Sesamum indicum*), focusing on the gene number, gene length, average CDS length, and the average exon number. We found consistent distribution trends across these species (Supplementary Table S5). These results demonstrate the reliability and accuracy of the gene prediction, reflecting a high level of confidence in the annotation process.

## Supplementary information

Supplementary Table S1-S5

## Data Availability

The genome assembly of *Utricularia aurea* is available in GenBank under accession number JBPYBC000000000 (https://www.ncbi.nlm.nih.gov/datasets/genome/GCA_056322005.1/), linked to BioProject PRJNA1289348 (https://www.ncbi.nlm.nih.gov/bioproject/?term=PRJNA1289348) including SRA database with accession number SRR34843000^33^, SRR34844152^34^, SRR34844153^35^, SRR34844667- SRR34844670^36–39^. All raw sequencing data and assembled sequences are publicly accessible. The assembled genome and annotation results have been deposited in the figshare database (10.6084/m9.figshare.29833016)^41^.

## Code availability

All data analyses were performed using standard bioinformatic tools that can freely obtained to the public, with parameters and software versions are detailed in the Methods section and Supplementary Table S1. All software and code utilized in this study are publicly accessible.

## References

[1] Taylor, P. G. *The Genus Utricularia: A Taxonomic Monograph*. First edn, Vol. 43 (Royal Botanic Gardens, Kew, 1989).

[2] Król, E. *et al*. Quite a few reasons for calling carnivores “the most wonderful plants in the world. *Annals of Botany* **109**, 47–64, 10.1093/aob/mcr249 (2011). 21937485 10.1093/aob/mcr249 PMC3241575

[3] Poppinga, S., Weisskopf, C., Westermeier, A., Masselter, T. & Speck, T. Fastest predators in plant kingdom: Functional morphology and biomechanics of suction traps found in the largest genus of carnivorous plants. *AoB Plants* **8**, plv140, 10.1093/aobpla/plv140 (2015). 26602984 10.1093/aobpla/plv140 PMC4717191

[4] Rutishauser, R. & Isler, B. Developmental Genetics and Morphological Evolution of Flowering Plants, Especially Bladderworts (*Utricularia*): Fuzzy Arberian morphology complements classical morphology. *Annals of Botany* **88**, 1173–1202, 10.1006/anbo.2001.1498 (2001).

[5] Ibarra-Laclette, E. *et al*. Architecture and evolution of a minute plant genome. *Nature* **498**, 94–98, 10.1038/nature12132 (2013). 23665961 10.1038/nature12132 PMC4972453

[6] Frantiek, Z. *et al*. The smallest angiosperm genomes may be the price for effective traps of bladderworts. *Annals of Botany* **134**, 1131–1138, 10.1093/aob/mcae107 (2024). 39012023 10.1093/aob/mcae107 PMC11688529

[7] Lan, T. *et al*. Long-read sequencing uncovers the adaptive topography of a carnivorous plant genome. *Proceedings of the National Academy of Sciences* **114**, E4435–E4441, 10.1073/pnas.1702072114 (2017). 10.1073/pnas.1702072114 PMC5465930 28507139

[8] Silva, S. *et al*. The Terrestrial Carnivorous Plant Utricularia reniformis Sheds Light on Environmental and Life-Form Genome Plasticity. *International Journal of Molecular Sciences* **21**, e3, 10.3390/ijms21010003 (2020). 10.3390/ijms21010003 PMC6982007 31861318

[9] Liu, B. *et al*. Estimation of genomic characteristics by analyzing k-mer frequency in de novo genome projects. *Quantitative Biology* **35**, 62–67, 10.48550/arXiv.1308.2012 (2013).

[10] Ranallo-Benavidez, T. R., Jaron, K. S. & Schatz, M. C. GenomeScope 2.0 and Smudgeplot for reference-free profiling of polyploid genomes. *Nature communications* **11**, 1432, 10.1038/s41467-020-14998-3 (2020). 32188846 10.1038/s41467-020-14998-3 PMC7080791

[11] Wick, R. R., Judd, L. M. & Holt, K. E. Performance of neural network basecalling tools for Oxford Nanopore sequencing. *Genome biology* **20**, 129, 10.1101/543439 (2019). 31234903 10.1186/s13059-019-1727-y PMC6591954

[12] Cali, D. S., Kim, J. S., Ghose, S., Alkan, C. & Mutlu, O. Nanopore sequencing technology and tools for genome assembly: computational analysis of the current state, bottlenecks and future directions. *Brief Bioinform* **20**, 1542–1559, 10.1093/BIB/BBY017 (2019). 29617724 10.1093/bib/bby017 PMC6781587

[13] Hu, J., Fan, J. P., Sun, Z. Y. & Liu, S. L. NextPolish: A fast and efficient genome polishing tool for long-read assembly. *Bioinformatics (Oxford, England)* **36**, 3210–3212, 10.1093/bioinformatics/btz891 (2019). 10.1093/bioinformatics/btz891 31778144

[14] Chen, S. F., Zhou, Y. Q., Chen, Y. R. & Gu, J. fastp: an ultra-fast all-in-one FASTQ preprocessor. *Bioinformatics* **34**, i884–i890, 10.1093/bioinformatics/bty560 (2018). 30423086 10.1093/bioinformatics/bty560 PMC6129281

[15] Langmead, B. & Salzberg, S. L. Fast gapped-read alignment with Bowtie 2. *Nature Methods* **9**, 357–359, 10.1038/nmeth.1923 (2012). 22388286 10.1038/nmeth.1923 PMC3322381

[16] Servant, N. *et al*. HiC-Pro: an optimized and flexible pipeline for Hi-C data processing. *Genome biology* **16**, 259, 10.1186/s13059-015-0831-x (2015). 26619908 10.1186/s13059-015-0831-x PMC4665391

[17] Burton, J. N. *et al*. Chromosome-scale scaffolding of de novo genome assemblies based on chromatin interactions. *Nature Biotechnology* **31**, 1119–1125, 10.1038/nbt.2727 (2013). 24185095 10.1038/nbt.2727 PMC4117202

[18] Wang, X. W. & Wang, L. GMATA: An Integrated Software Package for Genome-Scale SSR Mining, Marker Development and Viewing. *Frontiers in Plant Science* **7**, 1350, 10.3389/fpls.2016.01350 (2016). 27679641 10.3389/fpls.2016.01350 PMC5020087

[19] Gary, B. Tandem repeats finder: a program to analyze DNA sequences. *Nucleic Acids Research* **27**, 573–580, 10.1093/nar/27.2.573 (1999). 9862982 10.1093/nar/27.2.573 PMC148217

[20] Han, Y. & Wessler, S. R. MITE-Hunter: a program for discovering miniature inverted-repeat transposable elements from genomic sequences. *Nucleic Acids Res* **38**, e199, 10.1093/nar/gkq862 (2010). 20880995 10.1093/nar/gkq862 PMC3001096

[21] Bedell, J. A., Ian, K. & Warren, G. MaskerAid: a performance enhancement to RepeatMasker. *Bioinformatics* **16**, 1040–1041, 10.1093/bioinformatics/16.11.1040 (2000). 11159316 10.1093/bioinformatics/16.11.1040

[22] Keilwagen, J. *et al*. Using intron position conservation for homology-based gene prediction. *Nucleic Acids Research* **44**, e89–e89, 10.1093/nar/gkw092 (2016). 26893356 10.1093/nar/gkw092 PMC4872089

[23] Dobin, A. *et al*. STAR: ultrafast universal RNA-seq aligner. *Bioinformatics* **29**, 15–21, 10.1093/bioinformatics/bts635 (2013). 23104886 10.1093/bioinformatics/bts635 PMC3530905

[24] Haas, B. J. *et al*. Automated eukaryotic gene structure annotation using EVidenceModeler and the Program to Assemble Spliced Alignments. *Genome biology* **9**, R7, 10.1186/gb-2008-9-1-r7 (2008). 18190707 10.1186/gb-2008-9-1-r7 PMC2395244

[25] Pertea, M., Kim, D., Pertea, G. M., Leek, J. T. & Salzberg, S. L. Transcript-level expression analysis of RNA-seq experiments with HISAT, StringTie and Ballgown. *Nature Protocols* **11**, 1650–1667, 10.1038/nprot.2016.095 (2016). 27560171 10.1038/nprot.2016.095 PMC5032908

[26] Stanke, M., Diekhans, M., Baertsch, R. & Haussler, D. Using native and syntenically mapped cDNA alignments to improve de novo gene finding. *Bioinformatics* **24**, 637–644, 10.1093/bioinformatics/btn013 (2008). 18218656 10.1093/bioinformatics/btn013

[27] Lowe, T. M. & Eddy, S. R. tRNAscan-SE: a program for improved detection of transfer RNA genes in genomic sequence. *Nucleic Acids Res* **25**, 955–964, 10.1093/nar/25.5.955 (1997). 9023104 10.1093/nar/25.5.955 PMC146525

[28] Nawrocki, E. P. & Eddy, S. R. Infernal 1.1: 100-fold faster RNA homology searches. *Bioinformatics* **29**, 2933–2935, 10.1093/bioinformatics/btt509 (2013). 24008419 10.1093/bioinformatics/btt509 PMC3810854

[29] Griffiths-Jones, S. *et al*. Rfam: annotating non-coding RNAs in complete genomes. *Nucleic Acids Res* **33**, D121–124, 10.1093/nar/gki081 (2005). 15608160 10.1093/nar/gki081 PMC540035

[30] Lagesen, K. *et al*. RNAmmer: consistent and rapid annotation of ribosomal RNA genes. *Nucleic Acids Res* **35**, 3100–3108, 10.1093/nar/gkm160 (2007). 17452365 10.1093/nar/gkm160 PMC1888812

[31] Zdobnov, E. M. & Apweiler, R. InterProScan–an integration platform for the signature-recognition methods in InterPro. *Bioinformatics* **17**, 847–848, 10.1093/bioinformatics/17.9.847 (2001). 11590104 10.1093/bioinformatics/17.9.847

[32] McGinnis, S. & Madden, T. L. BLAST: at the core of a powerful and diverse set of sequence analysis tools. *Nucleic Acids Res* **32**, W20–W25, 10.1093/nar/gkh435 (2004). 15215342 10.1093/nar/gkh435 PMC441573

[33] *NCBI Sequence Read Archive* https://identifiers.org/ncbi/insdc.sra:SRR34843000 (2025). 1.

[34] *NCBI Sequence Read Archive* https://identifiers.org/ncbi/insdc.sra:SRR34844152 (2025). 2.

[35] *NCBI Sequence Read Archive* https://identifiers.org/ncbi/insdc.sra:SRR34844153 (2025). 3.

[36] *NCBI Sequence Read Archive* https://identifiers.org/ncbi/insdc.sra:SRR34844667 (2025). 4.

[37] *NCBI Sequence Read Archive* https://identifiers.org/ncbi/insdc.sra:SRR34844668 (2025). 5.

[38] *NCBI Sequence Read Archive* https://identifiers.org/ncbi/insdc.sra:SRR34844669 (2025). 6.

[39] *NCBI Sequence Read Archive* https://identifiers.org/ncbi/insdc.sra:SRR34844670 (2025). 7.

[40] *NCBI Assembly* https://www.ncbi.nlm.nih.gov/nuccore/JBPYBC000000000.1/ (2025).

[41] Yu, J. & Dong, H. Chromosome-level genome assembly of *Utricularia aurea* Lour., a canivorious higher plant with minute genome. *figshare*10.6084/m9.figshare.29833016 (2025). 10.1038/s41597-026-07285-1 PMC13280156 42009695

[42] Li, H. & Durbin, R. Fast and accurate long-read alignment with Burrows-Wheeler transform. *Bioinformatics* **26**, 589–595, 10.1093/bioinformatics/btp698 (2010). 20080505 10.1093/bioinformatics/btp698 PMC2828108

[43] Li, H. *et al*. The Sequence Alignment/Map format and SAMtools. *Bioinformatics* **25**, 2078–2079, 10.1093/bioinformatics/btp352 (2009). 19505943 10.1093/bioinformatics/btp352 PMC2723002

[44] Danecek, P. & McCarthy, S. A. BCFtools/csq: haplotype-aware variant consequences. *Bioinformatics* **33**, 2037–2039, 10.1093/bioinformatics/btx100 (2017). 28205675 10.1093/bioinformatics/btx100 PMC5870570

[45] Parra, G., Bradnam, K. & Korf, I. CEGMA: a pipeline to accurately annotate core genes in eukaryotic genomes. *Bioinformatics* **23**, 1061–1067, 10.1093/bioinformatics/btm071 (2007). 17332020 10.1093/bioinformatics/btm071

[46] Simão, F. A., Waterhouse, R. M., Ioannidis, P., Kriventseva, E. V. & Zdobnov, E. M. BUSCO: assessing genome assembly and annotation completeness with single-copy orthologs. *Bioinformatics* **31**, 3210–3212, 10.1093/bioinformatics/btv351 (2015). 26059717 10.1093/bioinformatics/btv351

[47] Rhie, A., Walenz, B. P., Koren, S. & Phillippy, A. M. Merqury: reference-free quality, completeness, and phasing assessment for genome assemblies. *Genome biology* **21**, 245, 10.1186/s13059-020-02134-9 (2020). 32928274 10.1186/s13059-020-02134-9 PMC7488777

